# What Are the Effects of Teaching Evidence-Based Health Care (EBHC)? Overview of Systematic Reviews

**DOI:** 10.1371/journal.pone.0086706

**Published:** 2014-01-28

**Authors:** Taryn Young, Anke Rohwer, Jimmy Volmink, Mike Clarke

**Affiliations:** 1 Centre for Evidence-based Health Care, Faculty of Medicine and Health Sciences, Stellenbosch University, Cape Town, South Africa; 2 South African Cochrane Centre, South African Medical Research Council, Cape Town, South Africa; 3 Community Health, Faculty of Medicine and Health Sciences, Stellenbosch University, Cape Town, South Africa; 4 All Ireland Hub for Trials Methodology Research, Queen’s University Belfast, Belfast, Northern Ireland; University of York, United Kingdom

## Abstract

**Background:**

An evidence-based approach to health care is recognized internationally as a key competency for healthcare practitioners. This overview systematically evaluated and organized evidence from systematic reviews on teaching evidence-based health care (EBHC).

**Methods/Findings:**

We searched for systematic reviews evaluating interventions for teaching EBHC to health professionals compared to no intervention or different strategies. Outcomes covered EBHC knowledge, skills, attitudes, practices and health outcomes. Comprehensive searches were conducted in April 2013. Two reviewers independently selected eligible reviews, extracted data and evaluated methodological quality. We included 16 systematic reviews, published between 1993 and 2013. There was considerable overlap across reviews. We found that 171 source studies included in the reviews related to 81 separate studies, of which 37 are in more than one review. Studies used various methodologies to evaluate educational interventions of varying content, format and duration in undergraduates, interns, residents and practicing health professionals. The evidence in the reviews showed that multifaceted, clinically integrated interventions, with assessment, led to improvements in knowledge, skills and attitudes. Interventions improved critical appraisal skills and integration of results into decisions, and improved knowledge, skills, attitudes and behaviour amongst practicing health professionals. Considering single interventions, EBHC knowledge and attitude were similar for lecture-based versus online teaching. Journal clubs appeared to increase clinical epidemiology and biostatistics knowledge and reading behavior, but not appraisal skills. EBHC courses improved appraisal skills and knowledge. Amongst practicing health professionals, interactive online courses with guided critical appraisal showed significant increase in knowledge and appraisal skills. A short workshop using problem-based approaches, compared to no intervention, increased knowledge but not appraisal skills.

**Conclusions:**

EBHC teaching and learning strategies should focus on implementing multifaceted, clinically integrated approaches with assessment. Future rigorous research should evaluate minimum components for multifaceted interventions, assessment of medium to long-term outcomes, and implementation of these interventions.

## Introduction

Evidence-based medicine (EBM) involves integrating clinical expertise acquired through clinical practice and experience, with patient values and current best evidence within the broader healthcare context [1], [2]. It is a systematic approach that includes lifelong self-directed learning in which caring for patients creates the need for important research-based information about clinical and other healthcare issues. As research evidence is constantly changing, healthcare professionals wishing to provide optimal care need to keep abreast of new developments to be able to offer interventions that work and eliminate the use of those shown to be harmful or ineffective [3]. Practicing EBM promotes critical thinking and typically involves five essential steps: first, converting information needs into answerable questions; second, finding the best evidence with which to answer the questions; third, critically appraising the evidence for its validity and usefulness; fourth, applying the results of the appraisal into clinical practice; and fifth, evaluating performance [4].

The concept of EBM has also been adopted by many allied healthcare professionals, and the Sicily statement of evidence-based practice [1] proposed that the concept of EBM be changed to evidence-based practice (EBP). In the healthcare setting, the term evidence-based health care (EBHC) is often used, as it is seen as beneficial for the entire healthcare team, allowing a more holistic, effective approach to the delivery of health care.

The importance of knowledge, skills and attitudes acquired through applying the principles of EBHC are emphasized in the Lancet commission report: *Education of health professionals for the 21^st^ century* [5], which highlights the need for healthcare professional training to be transformative. One of the key shifts of transformative learning aligns well with the steps of EBHC - the shift from memorization of facts to *“critical reasoning that can guide the capacity to search, analyze, assess and synthesize information for decision-making”* [5].

### Teaching and Learning EBHC

It is recommended that EBHC becomes a core component of the curriculum for all healthcare professionals, since learning the fundamentals of research and how to apply an evidence-based approach are essential for successful implementation of EBHC and subsequent improvement in the quality of health care [6].

**Figure 1:**
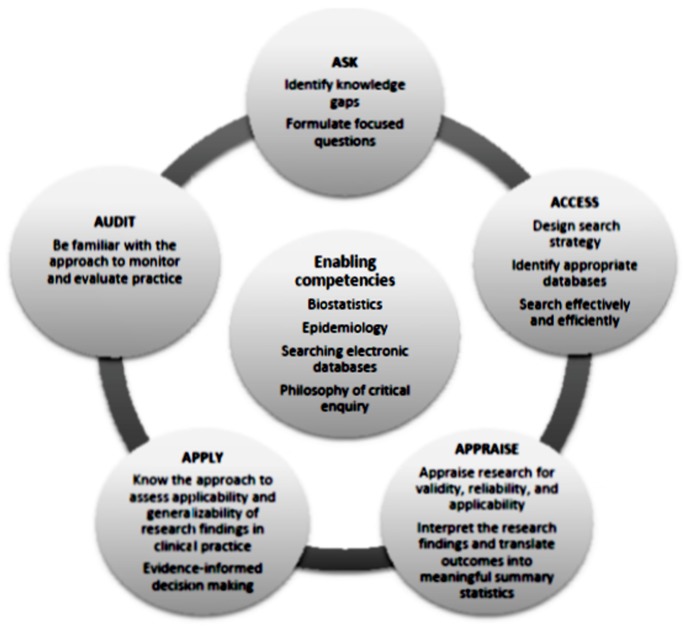
EBHC competencies.

Various learning and teaching strategies exist. Teaching can be done as standalone sessions or be integrated with clinical practice. It may include journal clubs, bed-side teaching, workshops, lectures, etc. Furthermore, it may be offered using face:face contact sessions, online learning or both, and can include both individual and group teaching and learning. The teaching approach may use directed learning or self-directed (problem-based) learning. The content of EBHC curricula is based on the five steps of EBHC and key competencies required to practice EBHC (Figure 1) also build on these steps [1], [7]. Expert teachers and facilitators pay a role in influencing learning and teaching in EBHC [8].

Educational activities can impact on EBHC knowledge, skills, attitudes and practice and, ultimately, the quality of health care and outcomes for patients. This links to Kirkpatrick’s four recommended levels (reaction, learning, behavior and results) for assessing training programme outcomes [9]. Validated tools to assess knowledge and skill acquisition exist and have been widely used [10], but similar, validated tools to determine the extent to which attitudes change after an educational intervention are lacking. Most studies reporting change in attitude or behavior rely on student self-reports as measurement tools, but this is not a reliable method for measuring long-term changes in attitude or effects on patient outcomes [10], [11].

In the clinical setting the ultimate goals are behavior change and improved patient outcomes [12]–[14] and these measures should ideally be used to assess whether teaching and learning of EBHC have been successful. A framework suggested by Michie et al. [15] describes a *“behaviour change wheel”*, where capability, opportunity and motivation are the three essential conditions that influence behaviour. In applying this to EBHC, capability could be viewed as a specific set of knowledge and skills; opportunity would refer to the available resources; and motivation would come from the individual attitudes towards EBHC.

Evaluation of EBHC-related educational activities should take into account the unique features of health professional education. This should include the different settings where learning takes place (bed-side, clinical, remote, outpatient, ambulatory), the background and learning style of the learners, the delivery format of courses (for example, large lectures, small groups, one-to-one tuition), and the structure of courses within the larger curriculum (stand-alone courses, integrated teaching) [16].

### Why It is Important to Do This Overview

Various systematic reviews assessing different teaching approaches, and including different target populations, have examined the effects of teaching EBHC. This overview synthesized evidence from systematic reviews of studies of teaching EBHC at undergraduate or post-graduate level and the impact of this teaching on EBHC competencies. We took a systematic approach to gather, evaluate and organize the review-level evidence on teaching EBHC, taking into consideration factors such as type of teaching and target audience, in order to improve access to the evidence and to inform EBHC teaching approaches. The objectives were to assess the effects of teaching EBHC to undergraduate and postgraduate health professionals.

## Methods

### Criteria for Considering Systematic Reviews for Inclusion

Systematic reviews which included randomized trials, quasi-randomized trials, controlled before-and-after studies and interrupted time series were eligible. Systematic reviews were defined as those that had predetermined objectives, predetermined criteria for eligibility, searched at least two data sources, of which one needed to be an electronic database, and performed data extraction and risk of bias assessment. Reviews were eligible if they evaluated any educational intervention (defined as a coordinated educational activity, of any medium, duration or format) to teach any component of EBHC (defined as the process of asking questions, accessing (literature searching), assessing and interpreting evidence by systematically considering its validity, results and relevance to ones’ own work) compared to no intervention or a different strategy in both undergraduate and postgraduate health professionals (at both student and professional levels). All health professionals including doctors, dentists, nurses, occupational therapists, physiotherapists, dieticians, audiologists, mental health professionals, psychologists, counsellors, and social workers were considered. Outcomes of interest were EBHC knowledge, skills, attitudes and practice as well as health outcomes.

### Search Methods for Identification of Systematic Reviews

A search for systematic reviews was conducted using a variety of electronic sources including *The Cochrane Library* (April 2013), *The Campbell Library* (April 2013), MEDLINE (April 2013), SCOPUS, the Educational Resource Information Center (ERIC), the Cumulative Index to Nursing and Allied Health Literature (CINAHL) (June 2013), and BEME. No language restrictions were used. Search terms included the following (modified appropriately for the various resources).

1. meta-analysis.mp,pt. OR review.pt OR systematic review.tw.
2. Teaching/OR teach$.mp OR Education/OR educa$.mp OR learn$ OR instruct$ OR medical education.
3. Evidence Based Practice/OR evidence based pract$.mp OR Evidence Based Health Care.mp OR Evidence Based Medicine.mp OR EBM.mp.

Experts in the field were contacted and reference lists of included reviews were checked to identify further potential reviews for inclusion [17].

### Systematic Review Selection, Data Collection, Quality Assessment and Analysis

Two authors (TY and AR) independently assessed eligibility of potentially relevant articles, extracted data and assessed quality of included systematic reviews. Titles, abstracts and descriptor terms of the records retrieved by the electronic searches were screened independently for relevance, based on the participant characteristics, interventions, and study design. Full text articles were obtained of all selected abstracts, as well as those where there was disagreement with respect to eligibility, to determine final selection. Differences in opinion were resolved by discussion.

Data were extracted independently using a predefined and piloted data extraction form. Data extracted included: the key characteristics of the systematic reviews, including information about the objectives; participant characteristics; intervention features including content, learning outcomes, teaching strategies, intervention intensities (frequency and duration); setting; outcomes assessed and instruments used to assess outcomes (including information regarding their reliability and validity); comparisons performed and results.

Using guidance from The Cochrane Collaboration [18], the quality of the included reviews was assessed. We aimed to discuss differences in quality between reviews, and use the review quality assessment to interpret the results of reviews synthesized in this overview. Quality of the reviews was not used as inclusion criteria, providing that it met the definition of a systematic review, as set out above. The methodological quality of each included systematic review was assessed using the AMSTAR (A MeaSurement Tool to Assess Reviews) instrument [19], which has been shown to have good face and content validity. AMSTAR assesses the degree to which review methods avoided bias by evaluating the methods reported against 11 distinct criteria. Each item on AMSTAR is rated as yes (clearly done), no (clearly not done), can’t answer, or not applicable. For all items, except item 4 (which relates to the exclusion of grey literature), a rating of ‘yes’ is considered adequate. For item 4, a rating of ‘no’ (that is, the review did not exclude unpublished or grey literature) is considered adequate. A review that adequately meets all of the 11 criteria is considered to be a review of the highest quality. Summary scores are typically classified as 3 or lower (low quality), 4 to 7 (medium quality) and 8 to 11 (high quality) [19].

Where there were discrepancies or data queries related to included studies within the systematic reviews, we searched for and reviewed the data that had been reported in the source article for the included study. We resolved differences by discussion and consensus.

We planned to report the effects of strategies to teach EBHC using relevant measures of effect and related 95% confidence intervals. However, as most findings were poorly reported, with many reviews not reporting effect sizes, we reported a descriptive summary of review findings taking into consideration the participants, educational interventions, comparisons and outcomes assessed, and reported effect measures that were available. The conceptual framework used in this overview aimed to clarify “what works for whom under which circumstances and to what end” (Table 1) [20].

**Table 1:** Conceptual framework for data synthesis [20].

**What works?**	Learning objectives, interventions, teaching methods
**For Whom?**	Learners targeted by the intervention
**Under which Circumstances?**	Intervention setting, duration, frequency
**To what end?**	**Desired learner outcomes**
	Short term – knowledge and awareness
	Medium term – attitude
	Long term – practice

The protocol for the overview was developed and approved by Stellenbosch University Research Ethics Committee S12/10/262.

## Results

### Results of the Search

**Figure 2:**
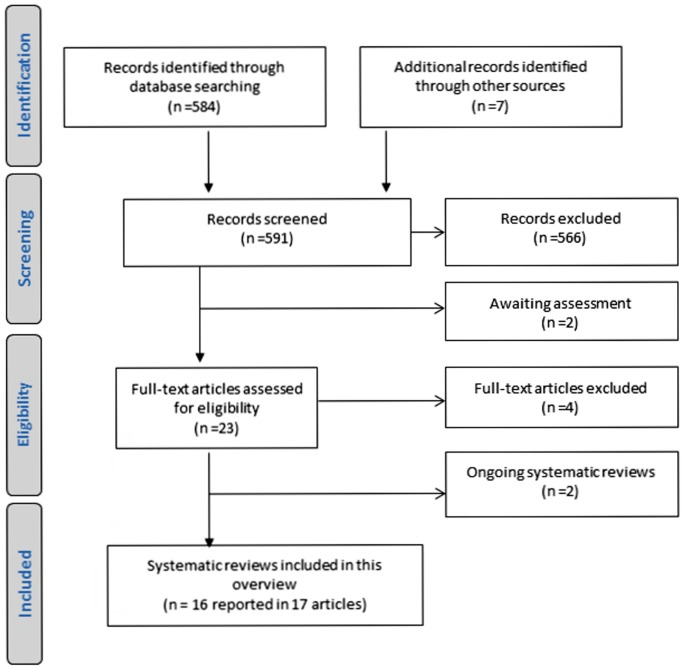
Flow diagram: Identification, screening and selection of systematic reviews.

Our electronic searches identified 584 article citations and a further seven records were found from other sources. After the initial screening of titles and abstracts, we retrieved 23 full text articles for formal eligibility assessment. Of these, we excluded four articles that did not meet the eligibility criteria (three were not systematic reviews and one did not assess teaching of EBHC) [21]–[24] (Table 2) and included 16 completed (reported in 17 articles) systematic reviews. Figure 2 details the process of selecting systematic reviews for inclusion using the ‘preferred reporting items for systematic reviews and meta-analyses’ (PRISMA) flow diagram [25].

**Table 2:** Excluded systematic reviews.

Study ID	Reason for exclusion
Alguire 1998 [21]	Not a systematic review
Malick 2010 [22]	Assessing assessment tools not effects of teaching interventions
Mi 2012 [23]	Not a systematic review
Werb 2004 [24]	Not a systematic review

### Description of Included Systematic Reviews

Fifteen published [26]–[40] and one unpublished [41] systematic review met the inclusion criteria (Tables 3A and 3B). One systematic review [27] was published in French. Furthermore, two ongoing systematic reviews [42], [43] are at the protocol development phase and two reviews are awaiting assessment [44], [45].

**Table 3:** Characteristics of included systematic reviews: Undergraduate and postgraduate.

Review ID	Types of participants	Interventions	Studiesincluded	Outcomes
Audet1993 [27]	Residents; UG medical students	Journal clubs; Weekly lectures; Once-offsessions; Biostatistics module	3 RCT; 5 CT;1 BA	Increased knowledge; Reading habits; Critical appraisal skills
Baradaran2013 [41]	Medical students (from 1st tofinal year); Clinical clerks; Interns	EBM lectures; EBM workshops; Integratedteaching of EBM; Online teaching of EBM	10 RCT; 5 CT;7 CBA; 4 BA	EBM knowledge; EBM skills; EBM behaviour; Critical appraisal skills; EBM attitude
Deenadayalan2008 [29]	UG, graduates, PG and clinicians	Journal clubs	3 RCT; 2 CT;2 BA	Reading habits; Critical appraisal skills; Knowledge of current medical literature; Research methods; Statistics
Harris2011 [33]	UG; PG	Journal clubs in different formats	2 RCT; 2 CT;5 BA	Change in reading behaviour; Confidence in critical appraisal; Demonstrated knowledge and critical appraisal skills; Ability to apply findings to clinical practice
Horsley2011 [35]	Interns in Internal Medicine,Health care professionals	Journal club supported by a half-dayworkshop; critical appraisal materials,list serve discussions and articles;Half-day workshop based on a CriticalAppraisal Skills Programme	3 RCT	Knowledge scores; Critical appraisal skills
Hyde2000 [36]	Medical students; Residents;Midwives; Intern doctors; qualifieddoctors, managers and researchers	Critical appraisals skills using Tutorial,Workshop, Lecture, Seminar, Study dayor Journal club	1 RCT; 8 CT;7 BA	Skills; Knowledge; Behaviour; Attitude
Ilic2009 [37]	UG/PG medical students orunder/PG allied healthprofessionals	Half day workshop; 7 week-2hour EBPworkshop; Multimedia package;Supplemented EBP teaching (directedvs. self-directed); Tutorials	3 RCT;3 CT	EBP competency; EBP knowledge, skills and behaviour; Critical appraisal skills; Formulating questions; Searching skills
Norman1998 [38]	UG medical residentsor residents	Undergraduate: EBM teaching ininternal medicine clerkship (part ofcourse credit); Residents: Variation ofjournal club format	2 RCT;8 CT	Knowledge and skills; Self-reported use of the literature
Taylor2000 [39]	Medical students and newlyqualified physicians	Educational interventions ranging froma total of 180 min over a 1-weekperiod to 16 h over the period of a year	1 RCT;8 CT	Knowledge of epidemiology/statistics; Attitudes towards medical literature; Ability to critically appraise and reading behaviour
Wong2013 [40]	Medical, Nursing and Physiotherapystudents; PG physiotherapy and UGoccupational therapy students	Mix of lecture-based and clinically-integratedEBP training covering different steps of EBP	2 CT;4 BA	Knowledge; Attitudes; Skills

Some of the systematic reviews were not limited to randomised controlled trials (RCT), controlled trials (CT) and controlled before-and-after studies (CBA) but also included other types of studies. For these reviews, we extracted data only on the findings from RCTs, CTs, CBAs and before after (BA) studies.

Included systematic reviews were published between 1993 and 2013. The first published in 1993, 6 more until 2006, and then 1 to 2 per year for the last seven years. One systematic review focused on undergraduate students [41], nine on both undergraduates and postgraduates [27], [29], [33], [35]–[40] and six on postgraduates only (including continuing professional development (CPD)) [26], [28], [30]–[32], [34].

The reviews evaluated many different educational interventions of varying duration, frequency and format (lectures, tutorials, journal clubs, workshops, online courses and integrated methods) to teach various components of EBHC (Tables 3 and 4). We categorized interventions into single interventions (SI) covering a workshop, journal club, lecture or e-learning, and multifaceted interventions (MI) where a combination of strategies had been assessed (e.g. lectures, tutorials, e-learning, journal clubs, etc.). The reviews also assessed a range of outcomes with a focus in many instances on acquisition of critical appraisal skills. Outcome assessment tools used varied considerably within and between systematic reviews.

**Table 4:** Characteristics of included systematic reviews: Postgraduate and continuing professional development.

POSTGRADUATE AND CONTINUING PROFESSIONAL DEVELOPMENT				
Review ID	Types of participants	Interventions	Studies included	Outcomes
Ahmadi2012 [26]	Residents	EBM teaching; Journal club	2 RCT;5 BA	EBM knowledge, EBM attitude, participants’ satisfaction; Critical appraisal knowledge, knowledge of EBM, knowledge of statistics and study design, self-assessed skills, research productivity, participants’ satisfaction
Coomarasamy2004 [28]	PG and healthcare professionalsattending continuing medicaleducation activities	Postgraduate EBM or critical appraisalteaching compared to control or baselinebefore teaching	4 RCT; 9 CT;10 BA	Knowledge, critical appraisal skills, attitude and behaviour
Ebbert2001 [30]	PG students	Journal club (small-group meeting todiscuss one or more journal articles)	2 RCT; 2 CT;1 BA	Critical appraisal skills, reading habits, knowledge of clinical epidemiology and biostatistics, use of medical literature in clinical practice
Flores Mateo2007 [31]	PG healthcare workers	Workshops; Multifaceted interventions;Internet-based intervention; Journal club(most common); Course and clinicalpreceptor; Educational presentation;Literature search course; Seminars	10 RCT; 6CT; 8 BA	EBM knowledge; EBM skills; EBM behaviour; EBM attitudes; Therapy supported by evidence
Green1999 [32]	Residents	Teaching critical appraisal skills usingseminars, multifaceted interventionsincluding seminars and journal clubs	1 RCT; 4CT; 2 BA	Residents’ knowledge of clinical epidemiology and critical appraisal; Students’ self-reported EBM behaviour
Horsley2010 [34]	Residents; Doctors, nurses,allied health professionals;Occupational healthphysicians	Lecture and input from librarian; Livedemonstrations, hands on practicesessions; Didactic input, hands-onpractice; Questionnaire with writteninstructions and examples	3 RCT;1 CT	Quality of questions; Increased success of answering questions; Knowledge-seeking practices; Self-efficacy; Types of questions generated

RCT – Randomized Controlled Trial.

CT – Controlled Trial.

CBA – Controlled Before After study.

BA – Before After study.

PG – Postgraduate.

UG - Undergraduate.

Details of the characteristics of each included systematic review are presented in Tables S1 to S16. Details of the ongoing systematic reviews are presented in Table S17.

### Quality of Systematic Reviews

The methodological quality of included systematic reviews varied widely (Table 5). The median AMSTAR score was 5 with a range of 3 to 10. Only four of the 16 had a high AMSTAR score [30], [34]–[36] (Table 5). The key methodological aspects which scored poorly included lack of a comprehensive search, not providing a list of both included and excluded studies, inappropriate methods to combine studies, not using scientific quality appropriately in formulating conclusions, not assessing publication bias and not declaring conflicts of interest. In some instances, AMSTAR items were not reported and were assessed as unclear.

**Table 5:** AMSTAR scores of included systematic reviews.

CRITERIA	Ahmadi2012[26]	Audet1993[27]	Baradaran2013[41]	Coomarasamy2004[28]	Deenadayalan2008[29]	Ebbert2001[30]	Flores Mateo2007[31]	Green1999[32]	Harris2011[33]	Horsley2010[34]	Horsley2011[35]	Hyde2000[36]	Ilic2009[37]	Norman1998[38]	Taylor2000[39]	Wong2013[40]
Was an a priori design provided?	Y	Y	Y	Y	Y	Y	Y	Y	Y	Y	Y	Y	Y	Y	Y	Y
Was there duplicate study selectionand data extraction?	Y	?	?	N	?	Y	Y	N	Y	Y	Y	Y	N	N	?	N
Was a comprehensive literaturesearch performed?	N	N	Y	Y	Y	Y	N	N	Y	Y	Y	Y	N	N	N	N
Was the status of publication (i.e. greyliterature) used as an inclusion criterion?	?	N	?	?	?	N	Y	Y	?	N	N	N	?	?	N	?
Was a list of studies (included andexcluded) provided?	N	N	N	N	Y	Y	N	N	Y	Y	Y	Y	Y	N	N	N
Were the characteristics of the includedstudies provided?	Y	Y	Y	Y	Y	Y	Y	Y	N	Y	Y	Y	Y	Y	Y	Y
Was the scientific quality of the includedstudies assessed and documented?	N	Y	Y	N	Y	Y	Y	Y	?	Y	Y	Y	?	Y	Y	N
Was the scientific quality of the includedstudies used appropriately in formulatingconclusions?	N	Y	?	N	N	Y	N	?	N	Y	Y	Y	N	N	?	N
Were the methods used to combine thefindings of studies appropriate?	?	Y	N	?	Y	?	?	Y	Y	Y	Y	Y	Y	N	?	Y
Was the likelihood of publication biasassessed? (where relevant)	?	N	N	?	N	N	Y	N	N	N	N/A	Y	N	N	N	N
Was the conflict of interest stated?	N	N	N	N	N	N	Y	N	Y	Y	Y	N	N	N	N	Y
**AMSTAR scores**	3	6	4	3	6	8	6	4	6	10	10	10	4	3	4	4

Y – Yes; N – No; ? – Unclear; N/A – Not applicable.

### Effects of Various Educational Interventions

In many instances, the systematic reviews did not report effect sizes or significance tests. Outcomes were narratively reported as improved or not, and vote counting was used. The focus was on short term outcomes, such as knowledge and skills, and none of the reviews found studies which reported on practice outcomes.

#### Systematic review level findings

One high quality review assessing interventions for improving frequency, quality and/or answerability of questions by healthcare professionals [34] reported that three of the four included studies, using mostly MI, showed improvements in question formulation in the short- to medium term. This improvement, assessed in one study, was however not sustained at one year. The authors of this review found no studies on interventions to increase the frequency or quality of questions generated explicitly and specifically within the context of reflective practice.

Four reviews, two high quality [35], [36] and two medium quality [27], [39], found that teaching critical appraisal improved participants knowledge on critical appraisal [27], [35], [36], [39], skill [27], [36], reading habit [27], [39] and attitude [36], [39]. Another review, which was judged to be of low quality, also found increased knowledge when teaching critical appraisal at undergraduate level [38] with a smaller increase in knowledge amongst residents.

Amongst postgraduates and healthcare professionals attending continuing medical education activities, a review of low quality [28] reported improved knowledge with both standalone and integrated teaching, while skills, attitudes and behaviour (changes in reading habits, choice of information resources as well as changes in management of patients and guidelines) improved with integrated methods. Another review of medium quality, amongst postgraduates [31] also found improved knowledge, skills and behaviour with workshops. Four reviews [29], [30], [32], [33], medium quality, assessed the effect of journal clubs amongst undergraduates and post graduates and found that they led to improved knowledge and reading behaviour [30], [33] however the included RCTs found no effect on critical appraisal skills [30], [32], [33].

One medium quality review [41] assessing a variety of teaching strategies for undergraduates, found improved knowledge, attitude and skills with e-learning compared to no intervention, no difference between e-learning and lectures, and improved knowledge and attitudes with MIs. Amongst residents, there was also no difference between e-learning and lectures [26]. Another medium quality review [40] assessed a MI amongst undergraduates and postgraduates consisting of a mix of lecture-based and clinically-integrated EBP training covering different steps of EBP and reported increased knowledge, attitude and behavior while another review [37], also of medium quality, found mixed results and no difference between directed and self-directed learning.

None of the reviews found evidence on process of care or patient outcomes.

#### Overlap between included systematic reviews

**Figure 3:**
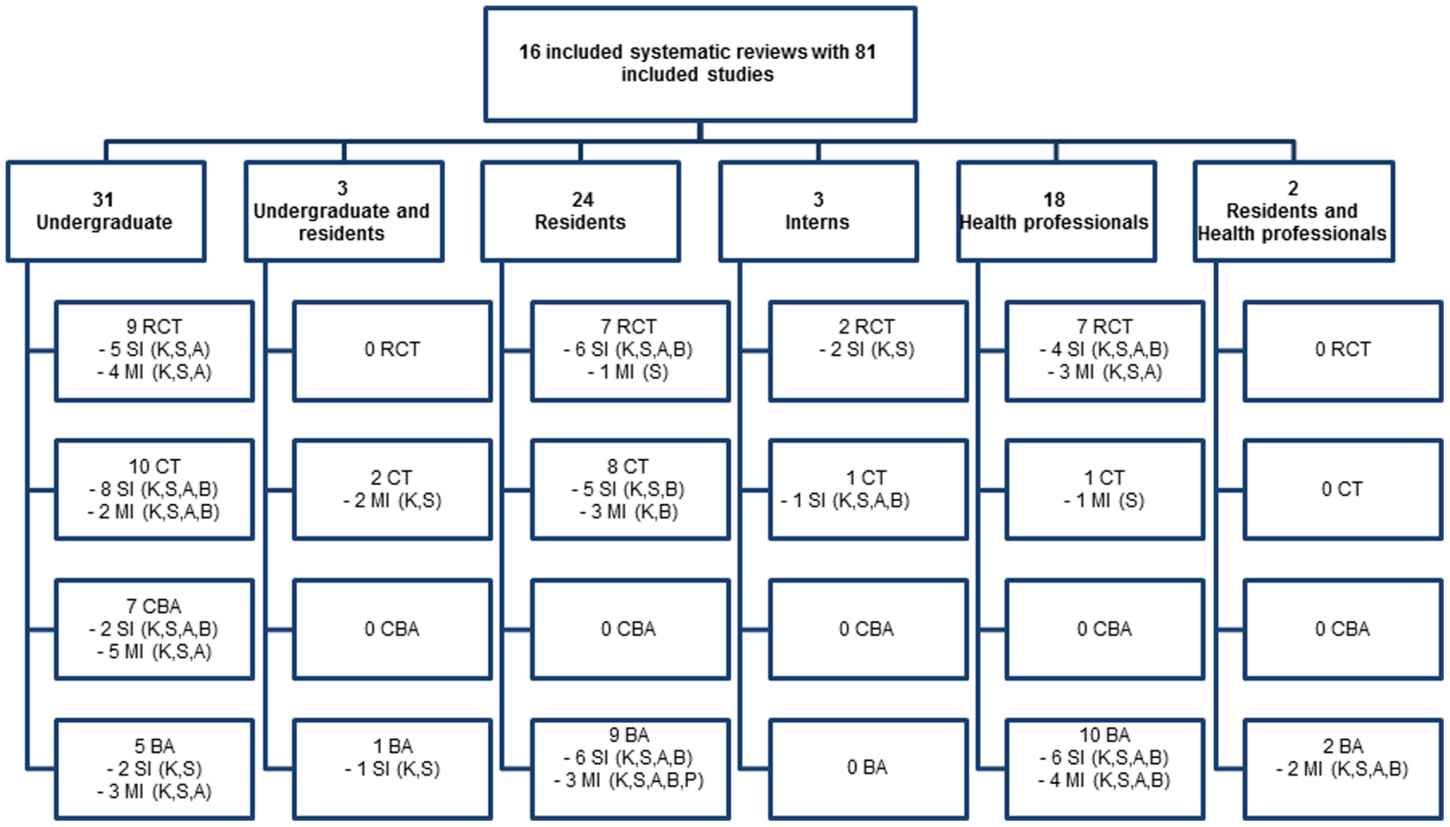
Summary of source studies included in the systematic reviews. K- Knowledge; S – Skills; A – Attitude; B – Behaviour; P – Practice; SI – Single intervention; MI – Multifaceted intervention; BA – Before After study; CBA – Controlled Before After study; CT – Controlled Trial; RCT – Randomized Controlled Trial.

We found considerable overlap in the studies included within the 16 systematic reviews (Table S18). Collectively, 171 studies were included in the reviews but these relate to a total of only 81 separate studies, of which 37 are included in more than one review. The breakdown of these studies by type of participant shows that 31 studies (9 RCTs, 10 CTs, 7 CBAs and 5 BAs) were amongst undergraduates, three studies (2 RCTs and 1 CT) were amongst interns, three studies (2 CTs, 1 BA) included undergraduates and residents, 24 studies (7 RCTs, 8 CTs and 9 BAs) were in residents, 18 studies (7 RCTs, 1 CT and 10 BAs) were in health professionals and two studies (2 BAs) included both residents and health professionals (Figure 3). As many of the source studies were included more than once (Table 5), and in an effort to organize and present a clear picture of the review level findings of the various educational interventions, and avoid double counting which would have given extra weight to findings from studies that had been used more than once, the following section provides a narrative summary of the findings from the 81 source studies as reported in the systematic reviews, and using the information provided on them within the reviews. This did not include the assessment of the methodological quality of these studies.

#### Findings from source studies

For undergraduate students, findings from the nine RCTs (sample size ranging from 77 to 238) indicated that MI, which included various combinations of strategies such as lectures, computer lab sessions, small-group discussions, journal clubs, use of real clinical issues, portfolios and assignments, presented over a few weeks, were more likely to improve knowledge, skills and attitudes compared to SI offered over a short duration or to no interventions. Twelve CTs (sample size ranging from 17 to 296) also found improved skill with MI. Some CTs found that SI had no effect on outcomes in the short term, while others found that searching skills and knowledge of appraisal improved when comparing interactive sessions with didactic sessions; and critical appraisal sessions with no interventions. The seven CBAs (sample size: 36 to 132 participants) found that knowledge and skills improved with MI (lectures, small group discussions, appraisal of various study types, real examples, computer lab sessions, feedback on appraisal) especially when measured over the few weeks after the MI. One CBA assessed a three month e-course and found improved knowledge, while two CBAs of short workshops that covered asking, acquiring and applying found improved knowledge, skills and attitude. The five BAs (sample size: 18 to 203 participants) also found improved skills after MI and improved knowledge and skills after a short workshop (3–4 days duration). In one BA, the MI included 18 weeks access to six online modules, plus supervised assignments in asking, acquiring, appraising various study types, and applying, linking to real patients. In another BA, it consisted of two sessions in EBM resources and appraising plus electronic exploratory notes, 6×2 hour small-group bedside sessions to exercise asking, self-searching, presenting critical appraisal topics in journal clubs, and developing EBM reports in portfolios.

Amongst interns, 2 RCTs (sample size: 55 to 237 participants) found no difference in knowledge and attitude towards EBM when comparing a face:face teaching session with access to e-learning modules. One CT (n = 30) assessing a short seminar, found no difference in the number of hours interns read per week, in confidence in evaluating articles, and critical appraisal, compared to no intervention.

For postgraduates and continuing professional development, seven RCTs (sample size: 10 to 441 participants) assessed mainly SI amongst residents. There were no significant differences in EBM knowledge and attitudes when comparing lecture-based teaching versus online modules in one trial (n = 61). Another RCT (n = 441) compared a monthly traditional journal club with a monthly internet journal club over eight months. Participation in the internet journal club was poor, even though it was a compulsory learning activity for all residents (18% in the internet group compared with 96% in the moderated group), and there was no significant difference in critical appraisal skills. A comparison of journal club versus standard conference (n = 44) found a significant increase in clinical epidemiology and biostatistics knowledge (reported p = 0.04), no change in critical appraisal skills (reported p = 0.09), no impact on articles read or read “completely” but more participants in the intervention group reported changes in reading behaviour and in the way they incorporated the literature into their practice (80% vs. 44%). Another RCT (n = 85) found no difference in clinical epidemiology and biostatistics knowledge and reading habits when journal club was led by faculty compared to being led by a chief medical resident. A comparison of informative lectures with librarian input on search question accuracy versus observed searches (without feedback from a librarian) (n = 10) found improved question formulation in the intervention group but with no statistical significance at six months. Results of the other two RCTs were not reported in the included systematic review.

Of the eight CTs amongst residents (sample size: 27 to 83 participants), one (n = 32) found no difference in reading habits, use of medical literature in clinical practice and critical appraisal skills when comparing journal club using critical appraisal techniques to traditional unstructured journal clubs. Another CT (n = 27) found no difference in pre-test versus post-test or between group scores for clinical epidemiology and biostatistics knowledge when comparing didactic sessions and journal clubs to no journal clubs. One further CT (n = 24) found no change in knowledge with journal club interventions. An eight hour seminar (n = 35) improved critical appraisal skills compared to no intervention (74% vs. 64%; p = 0.05) and a critical reading seminar with small group discussion (n = 83) significantly improved epidemiology and statistics knowledge (reported p = 0.019). Similarly, an EBM course (2 hours per week over 7 weeks) (n = 55) significantly improved skills. A CT of a MI of tutorials and one-on-one teaching (n = 34) found increased frequency of reading methods and results sections of articles, but no change in the hours reading; increased frequency of referral to an original article when faced with a clinical question; and significant improvement in critical appraisal skills and integration of results into patient decision making (reported p = 0.001). The result of the CT (n = 48), which assessed 10 workshops lasting 1–2 hours, was not reported in the systematic review.

Of the nine BAs (sample size: 8 to 73 participants) amongst residents, three evaluated MI and six SI. Results are available for two of the three BAs assessing MI. One (n = 8) assessed workshops on teaching critical appraisal skills as well as sessions on search skills prior to participating in weekly journal clubs. For each journal club session, residents identified articles relevant to a clinical question, critically appraised the articles, and presented a summarized critique. Comparing pre- and post-course scores, EBM knowledge and reading time increased significantly, but there were no differences in the number of literature searches and the number of articles read per week. The other BA (n = 14) evaluated small group sessions to teach library skills and journal club meetings and found an increase in EBM knowledge and number of literature searches. Of the BAs which assessed SI, one (n = 203) evaluated a EBM course delivered through small groups and found a significant increase in knowledge when comparing pre- and post-test scores, and two assessed journal clubs. One BA (n = 9) evaluated face:face monthly journal clubs over one year and found that EBM knowledge significantly improved while another (n = 29) assessed a quarterly journal club where participants reported improvement in skills, however, lowest perceived improvement occurred in the ability to critically appraise and assimilate evidence into clinical care.

Seven RCTs (sample size ranging 10 to 800 participants) assessed teaching interventions amongst practicing health professionals. One study (n = 81) assessed provision of articles, questions designed to guide critical appraisal, one-week listserv discussions on methodology of articles, and comprehensive methodological review of the article compared to just receiving articles and access to major journals, and found a significant increase in knowledge scores and critical appraisal skills. Another study (n = 148), evaluating a MI of a workshop in evidence-based public health, a newsletter, access to a specially designed information service, to relevant databases, and to an electronic discussion list, found a significant change in knowledge and behaviour but not in attitude; while another RCT (n = 392) evaluated a two-hour course and clinical preceptor (results not reported in systematic review). One RCT (n = 145) evaluated a three-hour workshop based on critical appraisal using problem based approaches which included didactic sessions, small group work, and a plenary session compared to no intervention and found a significant increase in knowledge scores, but no significant difference in critical appraisal skills. In assessing clinically integrated teaching, one RCT (n = 10) assessed EBM teaching rounds (daily ward rounds (except Mondays) focusing on development of searchable questions, literature search, critical appraisal, and application of evidence based on cases presented on clinical rounds) and found improvement in knowledge and behaviour. Two RCTs assessed interventions to enhance question formulation. One of these (n = 800) evaluated question formulation and live demonstrations, with hands-on practice sessions related to concepts of searching compared to no intervention and found a significant increase in the quality of questions phrased, increased success in answering questions and increased knowledge seeking practice. However, at 12 months, computer search logs revealed that search skills had eroded over time. The other study (n=52) compared a questionnaire with the addition of an explanation of the importance of proper question formulation, written instructions, and a diagrammatic example of how dimensional elements may be arranged, to a questionnaire without any instructions or examples and found that the intervention group was significantly more likely to explicitly describe patients (reported p = 0.028), comparisons (reported p = 0.014), and outcomes (reported p = 0.008).

One CT (n = 125) compared a four-day intensive EBM course which included didactic sessions, practical hands-on training in searching the Internet, training in critical appraisal and the provision of a flow chart of ways to consider relations between risk factors and disease and suggested search terms, to no flow chart provided or extra stimulants to use the flow chart. It found no significant differences in quality of question formulation, no differences between groups for mean time spent searching PubMed, and in retrieval of relevant articles. Of the 10 BAs (sample size: 12 to 1880 participants) amongst health professionals, three assessed workshops, one a study day, one a course and two assessed seminars. Knowledge and attitude increased with the workshops, while reading behaviour and critical appraisal skills increased with the study day. MI including EBM ward rounds led by clinical specialists and epidemiologists covering asking, searching, appraisal and summarising evidence on cases, and all weekly sessions based on problems encountered in clinical practice, found improved skills, attitude and behaviour. Two BAs included both residents and health professionals (sample size: 29 and 70 participants). One of these found improved skills after lectures and journal clubs while the other found no change in knowledge, skills and attitude after seminars followed by journal clubs.

## Discussion

The Sicily statement outlines that the content of EBHC curricula should be based on the five steps of EBHC [1]. This overview synthesized evidence from systematic reviews of studies of teaching EBHC at undergraduate or postgraduate level and the impact of this teaching on EBHC competencies. It took a systematic approach to gather, evaluate and organize the evidence that had been brought together in several systematic reviews [46], [47] on teaching EBHC, taking into consideration factors such as type of teaching and target audience, in order to improve access to the evidence and to inform EBHC teaching approaches.

### Summary of Main Results

Fifteen systematic reviews published between 1993 and 2013, one unpublished review and two on-going systematic reviews met the inclusion criteria. The systematic reviews evaluated many different educational interventions of varying duration, frequency and format (lectures, tutorials, journal clubs, workshops, online courses and integrated methods) to teach the various components of EBHC in a variety of settings. A range of outcomes were assessed with a focus in many systematic reviews on critical appraisal skills. Outcome assessment tools used varied considerably within and between systematic reviews. The 16 completed systematic reviews had considerable overlap in included studies and referred to a total of 81 source studies that had used one of the four study designs we pre-specified (RCTs, CTs, CBAs and BAs).

Most findings from the source studies were poorly reported in the included systematic reviews, without effect sizes or significance tests, and outcomes were often only described narratively as improved or not, with vote counting used. Consequently, and due to heterogeneity between studies, this overview reported results narratively. Findings from the studies amongst undergraduates were consistent. Multifaceted interventions (MI), with combinations of methods including lectures, computer lab sessions, small-group discussions, journal clubs, use of real clinical issues, and portfolios and assignments, were more likely to improve knowledge, skills and attitude compared to single interventions or no interventions. Amongst residents, these multifaceted clinically integrated interventions also improved critical appraisal skills and the integration of results into patient decision making, and improved knowledge, skills, attitude and behaviour amongst practicing health professionals. Considering SIs, for residents, EBHC knowledge and attitude were similar when comparing lecture-based teaching versus online modules. RCTs found that journal clubs increased clinical epidemiology and biostatistics knowledge and reading behavior, but not critical appraisal skills, whereas the CTs found no change in outcomes with journal clubs. Seminar/EBM courses improved critical appraisal skills and knowledge. Amongst practicing health professionals, an interactive online course with guided critical appraisal had a significant increase in knowledge and critical appraisal skills. Compared to no intervention, a short workshop using problem based approaches increased knowledge but not critical appraisal skills.

### Overall Completeness, Quality and Applicability of Evidence

The systematic reviews assessed a variety of educational interventions evaluated in many different settings and populations. Despite the notion that there is a lack of RCTs on educational interventions [20], the systematic reviews in this overview included 25 RCTs and a further 22 CTs. These studies had been conducted in high-income countries, and were published between 1984 and 2011. Outcome assessment methods ranged from validated tools [10] to those based on self-reports of participants. The content of some interventions, especially the single interventions, focused on critical appraisal which only covers part of the recommended EBHC curricula [1]. Multifaceted integrated interventions were more likely to include the application in patient decision making and how this can be implemented is being explored in ongoing research.

The focus of the systematic reviews was on EBHC knowledge, skills, attitudes and behaviour as outcomes, especially in the short term, and not assessing practice outcomes. These outcomes however were in line with three of the four recommended Kirkpatrick’s levels (reaction, learning and behaviour), which are widely accepted for assessing training programme outcomes [9]. It is important to be mindful that patient health outcomes, the fourth Kirkpatrick level, are influenced by many different factors of which health professional behaviour is only one component [48]. Glasziou and Haynes [49] outline several factors which influence translation of evidence to action. This starts with healthcare professionals being aware of the best evidence and accepting this evidence. Next, a decision needs to be made regarding the applicability of the evidence to the local setting and whether a particular intervention is available and can be implemented by healthcare professionals. As habits take time to change, high quality evidence, may not always be adopted by practitioners for translation into practice. Furthermore, patients may not agree to certain treatment approaches and even if they do, may not adhere to them. Considering the multitude of factors impacting on practice outcomes, teaching EBHC could conceivably impact on practitioners’ EBHC knowledge, skills, attitudes and behaviour, without necessarily influencing practice. This makes it difficult to design robust studies of appropriate sample size [50] and difficult to assess and attribute improved health outcomes to any single factor [48].

The methodological quality of the included systematic reviews varied. Most did not conduct a comprehensive literature search, did not report on both included and excluded studies, did not use the scientific quality of the included studies appropriately in formulating conclusions and did not assess for publication bias. Furthermore, the findings for the source studies, which were generally of small sample size, were generally poorly reported in the systematic reviews. In many instances, the reviews did not report effect sizes and results from significance tests, and reported summarised results narratively and in tabular format [20]. When we compared the information on studies that were included in more than one systematic review, there were discrepancies in data extracted and we obtained the original reports of these studies for the correct information. We found discrepancies in number of participants, outcomes reported, and the type of study design. Collectively, though, as presented in this overview, the included systematic reviews do give a good representation of studies that have assessed the effects of various educational interventions for teaching EBHC over the last two decades.

### Potential Biases in the Overview Process

Overviews of systematic reviews have been criticised for lack of methodological rigor, especially related to inadequate searching, bias in review selection, and lack of assessment of methodological quality of included reviews [47], [51], [52]. Drawing on methodology to conduct rigorous systematic reviews, the methods followed for this overview aimed to reduce selection, language, publication and indexing biases [18], [47]. We followed a pre-specified protocol. A comprehensive search, without language limitations, was conducted in various electronic databases, and we searched for on-going and unpublished systematic reviews. Additional searches were conducted to resolve discrepancies related to the studies included in the systematic reviews. We did not conduct additional searches for studies published after 2011. Two reviewers independently applied pre-defined eligibility criteria to select systematic reviews for inclusion, extracted data and evaluated the methodological quality of each included systematic review. PRISMA reporting guidelines were followed [25].

### Agreements and Disagreements with Other Studies or Reviews

Khan and colleagues [53] assessed evidence on interventions for changing clinician behaviour, educational effectiveness of CPD, and effective learning of EBM conclusions. Based on educational evidence, theory and principles Khan proposed a hierarchy of teaching and learning methods for EBM. Findings of this overview resonate with Khan’s [53] hierarchy of EBHC teaching and learning activities - *“Level 1, interactive and clinically integrated activities; Level 2(a), interactive but classroom based activities; Level 2(b), didactic but clinically integrated activities; and Level 3, didactic, classroom or standalone teaching.”*

## Conclusions

### Implications for Practice

EBHC competencies are necessary for providing high quality healthcare. Teaching and learning strategies to enhance these competencies need to focus on implementing multifaceted clinically integrated approaches with assessment.

### Implications for Research

Systematic reviews and robust RCTs are both useful in assessing health professional education strategies [54]. Future studies and systematic reviews should focus on minimum components for multifaceted interventions, assessment of EBHC knowledge, attitude, skills and behaviour in the medium to long term, using validated assessment tools [10], and how best to implement these interventions. Further evaluation should consider the effectiveness of e-learning and the influence of various teaching and learning settings and the context within which teaching takes place. It is important that future research carefully considers the questions to be addressed and refines these, based on existing evidence from systematic reviews to avoid unnecessary duplication [55], [56]. Adherence to rigorous methodological approaches [54] and good reporting practices [25], [54] are important to ensure a contribution to evidence informed decisions on the teaching and learning of EBHC.

## Supporting Information

Table S1 **Characteristics of included systematic review Ahmadi 2012.**
(DOCX)

Table S2 **Characteristics of included systematic review Audet 1993.**
(DOCX)

Table S3 **Characteristics of included systematic review Baradaran 2013.**
(DOCX)

Table S4 **Characteristics of included systematic review Coomarasamy 2004.**
(DOCX)

Table S5 **Characteristics of included systematic review Deenadayalan 2008.**
(DOCX)

Table S6 **Characteristics of included systematic review Ebbert 2001.**
(DOCX)

Table S7 **Characteristics of included systematic review Flores-mateo 2007.**
(DOCX)

Table S8 **Characteristics of included systematic review Green 1999.**
(DOCX)

Table S9 **Characteristics of included systematic review Harris 2011.**
(DOCX)

Table S10 **Characteristics of included systematic review Horsley 2010.**
(DOCX)

Table S11 **Characteristics of included systematic review Horsley 2011.**
(DOCX)

Table S12 **Characteristics of included systematic review Hyde 2000.**
(DOCX)

Table S13 **Characteristics of included systematic review Ilic 2009.**
(DOCX)

Table S14 **Characteristics of included systematic review Norman 1998.**
(DOCX)

Table S15 **Characteristics of included systematic review Taylor 2000.**
(DOCX)

Table S16 **Characteristics of included systematic review Wong 2013.**
(DOCX)

Table S17 **Characteristics of ongoing systematic reviews.**
(DOCX)

Table S18 **Matrix of included systematic reviews and the studies included in each.**
(DOCX)

Checklist S1 **PRISMA checklist.**
(DOC)
