# Supplementary material for: What Are the Effects of Teaching Evidence-Based Health Care (EBHC)? Overview of Systematic Reviews
Source: PLoS One. 2014 Jan 28;9(1):e86706. doi: 10.1371/journal.pone.0086706 (PMC3904944; doi:10.1371/journal.pone.0086706)
Supplement: Table S1 — Characteristics of included systematic review Ahmadi 2012. (DOCX) [file pone.0086706.s001.docx]

## Table S1. CHARACTERISTICS OF INCLUDED SYSTEMATIC REVIEW AHMADI 2012

|  | What the review authors searched for | What the review authors found |
| --- | --- | --- |
| Studies | Systematic review including RCT's, non-randomised comparisons, before-after studies | 7 studies: 1 RCT; 3 uncontrolled before and after studies; 3 surveys 8 studies: 1 RCT; 3 uncontrolled before and after studies; 3 surveys and 1 observational studies |
| Participants | Surgical residents | |
| Interventions | Two reviews- one on EBM teaching and another on Journal club | |
| Comparisons |  | Only reported for RCTs |
| Outcomes | 1. EBM knowledge, EBM attitude, participants' satisfaction 2. Critical appraisal knowledge, knowledge of EBM, knowledge of statistics and study design, self-assessed skills, research productivity, participants’ satisfaction | 1.EBM knowledge, EBM attitude, participants’ satisfaction  2. Critical appraisal knowledge, knowledge of EBM, knowledge of statistics and study design, self-assessed skills, research productivity, participants’ satisfaction |
| Date of the most recent search: July 2010 | | |
| **Limitations:** Search strategy not comprehensive; English language only; No risk of bias assessment for included studies | | |
| **Citation:** Ahmadi N, McKenzie ME, MacLean A, Brown C, Mastracci T, McLeod RS. Teaching Evidence-based Medicine to Surgery residents – Is journal club the best format? A Systematic Review of the literature. Journal of Surgical Education 2012:69; 91-100 | | |
