# Supplementary material for: What Are the Effects of Teaching Evidence-Based Health Care (EBHC)? Overview of Systematic Reviews
Source: PLoS One. 2014 Jan 28;9(1):e86706. doi: 10.1371/journal.pone.0086706 (PMC3904944; doi:10.1371/journal.pone.0086706)
Supplement: Table S2 — Characteristics of included systematic review Audet 1993. (DOCX) [file pone.0086706.s002.docx]

## Table S2. CHARACTERISTICS OF INCLUDED SYSTEMATIC REVIEW AUDET 1993

|  | What the review authors searched for | What the review authors found |
| --- | --- | --- |
| Studies | Did not specify - studies with at least 10 participants per group - controlled before and after studies; some RCT | 2 RCT (post-test only); 3 Controlled trials; 3 Controlled before-after studies; 1 Before-after study; 1 Cross-sectional study |
| Participants | Undergraduate and postgraduate medical students | Residents and Undergraduate medical students |
| Interventions | Critical appraisal teaching | Journal clubs; Weekly lectures; Once-off sessions; Biostatistics module |
| Comparisons | Not specified | |
| Outcomes | Knowledge in clinical epidemiology and biostatistics, reading habits, ability to critically appraise a scientific article | Increased knowledge; Reading habits; Critical appraisal skills |
| Date of the most recent search: Not clearly reported. Authors included studies published between 1980 and 1990 – The review was only published in 1993. | | |
| **Limitations:** Search limited to English and French studies; No report of publication status of studies; Only searched MEDLINE and FAMLI databases; Did not contact experts; No report of duplicate screening of abstracts; No list of excluded studies; Results described narratively, no measures of effect or Confidence interval reported; Authors used vote-counting based on direction of effect and statistical significance to summarise results | | |
| **Citation:** Audet N, Gagnon R, Ladouceur Rm Marcil M. L’enseignement de l’analyse critique des publications scientifiques médicales est-il efficace? Révision des etudes et de leur qualité méthodologique. Can Med Assoc J. 1993; 148(6):945-52 | | |
