# Supplementary material for: What Are the Effects of Teaching Evidence-Based Health Care (EBHC)? Overview of Systematic Reviews
Source: PLoS One. 2014 Jan 28;9(1):e86706. doi: 10.1371/journal.pone.0086706 (PMC3904944; doi:10.1371/journal.pone.0086706)
Supplement: Table S3 — Characteristics of included systematic review Baradaran 2013. (DOCX) [file pone.0086706.s003.docx]

## Table S3. CHARACTERISTICS OF INCLUDED SYSTEMATIC REVIEW BARADARAN 2013

|  | What the review authors searched for | What the review authors found |
| --- | --- | --- |
| Studies | Any comparative study – e.g. randomized controlled trials, non-randomized controlled trials, controlled before-after studies | 27 studies: 11 CBA’S; 10 RCT’S (2 cross over and 1 cluster); 6 Non-randomized controlled studies |
| Participants | Undergraduate medical students (defined as medical school students before entering residency programs) Excluded: postgraduate students | Medical students (from 1st to final year); Clinical clerks; Interns |
| Interventions | At least one educational intervention (def: coordinated educational activity of any medium, duration or format) to teach EBM. Excluded: Where content covered only teaching searching, biostatistics and epidemiology) | EBM lectures; EBM workshops; Integrated teaching of EBM; Online teaching of EBM |
| Comparisons | Not described | No teaching of EBM or different forms of teaching EBM |
| Outcomes | Students‘ knowledge, attitudes, skills, behaviours | EBM knowledge; EBM skills; EBM behaviour; Critical appraisal skills; EBM attitude |
| Date of the most recent search: May 2011 | | |
| **Limitations:** Did not contact experts; No list of excluded studies provided; No effect sizes and 95% confidence reported. Findings not substantiated by results; Meta-analysis conducted but not appropriate; Heterogeneity not explored; Differences between studies and results not analysed and described sufficiently | | |
| **Citation:** Baradaran HR, Amadi S-F, Ahmadi E. Teaching evidence-based medicine to undergraduate medical students: A systematic review and meta-analysis 2013 *(personal communication, not yet published, currently under peer review with BEME)* | | |
