# Supplementary material for: What Are the Effects of Teaching Evidence-Based Health Care (EBHC)? Overview of Systematic Reviews
Source: PLoS One. 2014 Jan 28;9(1):e86706. doi: 10.1371/journal.pone.0086706 (PMC3904944; doi:10.1371/journal.pone.0086706)
Supplement: Table S4 — Characteristics of included systematic review Coomarasamy 2004. (DOCX) [file pone.0086706.s004.docx]

## Table S4. CHARACTERISTICS OF INCLUDED SYSTEMATIC REVIEW COOMARASAMY 2004

|  | What the review authors searched for | What the review authors found |
| --- | --- | --- |
| Studies | Systematic review including RCT's, non-randomised controlled studies, before-after studies | Four RCT’s; Seven non-randomised controlled studies; 12 before and after studies |
| Participants | Postgraduate (health care professionals – not specified) and health care professionals attending continuing medical education activities (excluding undergraduates) | Postgraduate health care practitioners (did not specify in the report) |
| Interventions | Postgraduate EBM or critical appraisal teaching compared to control or baseline before teaching | Standalone EBM teaching – 18 studies and Integrated EBM teaching – 5 studies. Teaching methods included workshops, seminars, and journal clubs alone or in various combinations. Details of interventions – duration, learning outcomes, setting, etc not clearly reported. Integrated teaching focused on training in EBM components (such as question formulation, literature searching, and critical appraisal) in real  time clinical ward rounds or basing the EBM teaching sessions on encounters with patients on the wards and in clinics. Duration of interventions unclear. |
| Comparisons |  | Not reported |
| Outcomes | Participants' learning achievements: knowledge, critical appraisal skills, attitudes, behaviour; Patients' health gains | Knowledge, critical appraisal skills, attitude and behaviour; No patient health outcomes |
| Date of the most recent search: April 2004 | | |
| **Limitations:** Unclear whether search covered all languages; No methods detailed for independent selection of studies and data extraction; Description of excluded studies incomplete; No assessment of risk of bias of included studies; Results of individual studies not reported. | | |
| **Citation:** Coomarasamy A, Khan SK. What is the evidence that postgraduate teaching in evidence-based medicine changes anything? A systematic review. BMJ 2004; 329 and Coomarasamy A, Taylor R, Khan KS. A systematic review of postgraduate teaching in evidence-based medicine and critical appraisal. Medical Teacher. 2003; 25:1, 77-81 | | |
