# Supplementary material for: What Are the Effects of Teaching Evidence-Based Health Care (EBHC)? Overview of Systematic Reviews
Source: PLoS One. 2014 Jan 28;9(1):e86706. doi: 10.1371/journal.pone.0086706 (PMC3904944; doi:10.1371/journal.pone.0086706)
Supplement: Table S5 — Characteristics of included systematic review Deenadayalan 2008. (DOCX) [file pone.0086706.s005.docx]

## Table S5. CHARACTERISTICS OF INCLUDED SYSTEMATIC REVIEW DEENADAYALAN 2008

|  | What the review authors searched for | What the review authors found |
| --- | --- | --- |
| Studies | Experimental studies which directly and concurrently compared outcomes from journal club activities with outcomes from other forms of education; Quasi-experimental or comparative studies which assessed outcomes pre- and post-journal club inception | 3 RCT’s; 3 CT’s; 2 Cohort studies; 3 Curriculum reports; 5 Reports; 1 un blinded interventional study; 1 review of journal club; 1 feasibility study; 1 personal experience report; 1 pilot study |
| Participants | Health practitioners of any discipline | Undergraduates, graduates, postgraduates and clinicians from the following health disciplines were included: Obstetrics and Gynaecology; Clinical Epidemiology and Biostatistics; Internal Medicine; Psychiatry; Nursing; Geriatric Medicine |
| Interventions | Any form of journal club | |
| Comparisons | Any comparator | |
| Outcomes | Any outcome measure relating to journal club effectiveness, including knowledge, attitudes, skill acquisition, practice behaviours, satisfaction | Reading habits; Critical appraisal skills; Knowledge of current medical literature; Research methods; Statistics |
| Date of the most recent search: Not reported | | |
| **Limitations:** Only included articles in English language and where the full text was available; Authors /experts not contacted; Unclear whether publication status influenced inclusion (but authors only included studies of which the full text was available); Date of search unclear; Authors reported that they used the McMaster; University instrument to critically appraise studies and gave scores according to the 14 criteria. Criteria not reported – only score out of 14 for each included study; Did not report independent data extraction; Did not describe heterogeneity or the process of data synthesis | | |
| **Citation:** Deenadayalan Y, Grimmer-Somers K, Prior M, Kumar S. How to run an effective journal club: a systematic review. *Journal of Evaluation in Clinical Practice*. 2008;14: 898-911 | | |
