# Supplementary material for: What Are the Effects of Teaching Evidence-Based Health Care (EBHC)? Overview of Systematic Reviews
Source: PLoS One. 2014 Jan 28;9(1):e86706. doi: 10.1371/journal.pone.0086706 (PMC3904944; doi:10.1371/journal.pone.0086706)
Supplement: Table S6 — Characteristics of included systematic review Ebbert 2001. (DOCX) [file pone.0086706.s006.docx]

## Table S6. CHARACTERISTICS OF INCLUDED SYSTEMATIC REVIEW EBBERT 2001

|  | What the review authors searched for | What the review authors found |
| --- | --- | --- |
| Studies | RCT's, cohort studies, before-after studies, cross-sectional studies | 7 studies: 1 RCT; 3 Cohort studies; 1 Before and after study; 2 cross-sectional studies |
| Participants | Postgraduate physicians (interns and residents) training in any speciality or subspeciality | Postgraduate students (internal medicine, Paediatrics, Emergency medicine, Obstetrics and Gynaecology; Physical medicine and rehabilitation) |
| Interventions | Journal club (small-group meeting to discuss one or more journal articles) | |
| Comparisons | Not stated | No journal club, before journal club, Standard conference on topics in ambulatory care, traditional, unstructured journal club. |
| Outcomes | Critical appraisal skills, reading habits, knowledge of clinical epidemiology and biostatistics, use of medical literature in clinical practice, improved patient outcomes | Critical appraisal skills, reading habits, knowledge of clinical epidemiology and biostatistics, use of medical literature in clinical practice |
| Date of the most recent search: March 2000 | | |
| **Limitations:** Authors did not specify whether they had any language restrictions; Authors did not describe how they analysed results and how the variability between interventions influence results in the methods or results section, but mention in the discussion that “the lack of methodologically rigorous study designs and the apparent heterogeneity in the outcomes measured argued against pooling of the results” | | |
| **Citation:** Ebbert JO, Montori VM, Schultz HJ. The journal club in postgraduate medical education: a systematic review. Medical Teacher 2001: 23(5); 455-461 | | |
