# Supplementary material for: What Are the Effects of Teaching Evidence-Based Health Care (EBHC)? Overview of Systematic Reviews
Source: PLoS One. 2014 Jan 28;9(1):e86706. doi: 10.1371/journal.pone.0086706 (PMC3904944; doi:10.1371/journal.pone.0086706)
Supplement: Table S7 — Characteristics of included systematic review Flores-mateo 2007. (DOCX) [file pone.0086706.s007.docx]

## Table S7. CHARACTERISTICS OF INCLUDED SYSTEMATIC REVIEW FLORES-MATEO 2007

|  | What the review authors searched for | What the review authors found |
| --- | --- | --- |
| Studies | Systematic review including RCT's, non-randomised trials, Before-and-after studies | 24 studies: sample size ranging from 12 to 800 - 11 RCT’s; 5 non-randomized controlled trials; 8 Before-after studies |
| Participants | Postgraduate healthcare workers. Excluded - medical students and undergraduates; focused on prescribing practices; specific health problems; Theoretical reviews of different components of EBP; General continuing medical education; Testing the effectiveness of implementing guidelines; Evaluating teaching methods Using IT devices (PDA or computer-based reminder) | Postgraduate healthcare workers: Health care professionals; Medical interns; Physicians; Primary care residents; Medical research, managerial and nursing staff; Family medicine residents; Psychiatry residents; Emergency Medicine residents; Internal medicine residents; Medical residents; Experts in EBM and third year medical students; Public Health physicians; Surgeons; Paediatric residents; Occupational therapists; General practitioners |
| Interventions | Teaching EBP | Teaching EBP: Workshops; Multifaceted interventions; Internet-based intervention; Journal club (most common); Course and clinical preceptor; Educational presentation; Literature search course; Seminars |
| Comparisons | Not specified | Controls not described |
| Outcomes | Improved EBP knowledge, skills, attitudes and behaviour | EBM knowledge; EBM skills; EBM behaviour; EBM attitudes; Therapy supported by evidence |
| Date of the most recent search: December 2006 | | |
| **Limitations:** Search strategy did not include unpublished literature; authors did not contact experts in the fields for additional studies; No list of excluded studies; Risk of bias assessment not adequate for all included studies (quality assessment); Results reported selectively – only those results where authors were able to calculate an effect size were reported. Results of 14/24 included studies not reported. | | |
| Citation: Flores-Mateo G, Argimon JM. Evidence based practice in postgraduate healthcare education: A systematic review. BMC Health Services Research. 2007, 7:119 doi:10.1186/1472-6963-7-119 | | |
