# Supplementary material for: What Are the Effects of Teaching Evidence-Based Health Care (EBHC)? Overview of Systematic Reviews
Source: PLoS One. 2014 Jan 28;9(1):e86706. doi: 10.1371/journal.pone.0086706 (PMC3904944; doi:10.1371/journal.pone.0086706)
Supplement: Table S8 — Characteristics of included systematic review Green 1999. (DOCX) [file pone.0086706.s008.docx]

## Table S8. CHARACTERISTICS OF INCLUDED SYSTEMATIC REVIEW GREEN 1999

|  | What the review authors searched for | What the review authors found |
| --- | --- | --- |
| Studies | Not specified | 18 reports of EBM curricula (study design not specified) and 7 of these looked at the effectiveness of the curriculum and 5 of these had control group |
| Participants | Graduate medical education | Residents (7 in internal medicine, 3 in family medicine, 3 in obstetrics and gynaecology, 2 in paediatrics, 1 in surgery, 1 in emergency medicine and 1 inter-programme curriculum) |
| Interventions | EBM/critical appraisal curricula | Teaching critical appraisal skills: Journal club format in 13 studies; Integrated EBM teaching into clinical rotations: 2 studies; Integration of EBM in morning reports:2 studies; Comprehensive, program-wide curricular change: 1 study |
| Comparisons | Not specified | Not relevant for most studies, pretest-posttest design for most effectiveness studies |
| Outcomes | Curriculum development, curriculum objectives, curriculum formats, educational strategies, curriculum evaluation | Curriculum development; Curriculum objectives; Curriculum formats; Educational strategies; Curriculum evaluation (effectiveness) - Residents’ knowledge of clinical epidemiology and critical appraisal and Students’ self-reported EBM behaviour; Process evaluation; Satisfaction evaluation |
| Date of the most recent search: 1998 | | |
| **Limitations:** Selection criteria not sufficiently explicit and was very broad; Search strategy: did not contact authors, no mention of unpublished studies, only MEDLINE and ERIC searched; No list of excluded studies; No duplicate, independent selection of studies; Review focuses more on curriculum development and content than effectiveness. | | |
| Citation: Green ML. Graduate medical education training in clinical epidemiology, critical appraisal and evidence-based medicine: A critical review of curricula. Acad Med 1999;74(6):686-94 | | |
