# Supplementary material for: What Are the Effects of Teaching Evidence-Based Health Care (EBHC)? Overview of Systematic Reviews
Source: PLoS One. 2014 Jan 28;9(1):e86706. doi: 10.1371/journal.pone.0086706 (PMC3904944; doi:10.1371/journal.pone.0086706)
Supplement: Table S9 — Characteristics of included systematic review Harris 2011. (DOCX) [file pone.0086706.s009.docx]

## Table S9. CHARACTERISTICS OF INCLUDED SYSTEMATIC REVIEW HARRIS 2011

|  | What the review authors searched for | What the review authors found |
| --- | --- | --- |
| Studies | Systematic review including any quantitative or qualitative study evaluating journal clubs | 8 before and after studies; 6 questionnaire surveys; 1 observational study; 1 case control study; 1 controlled trial; 1 randomised controlled trial |
| Participants | Undergraduates in any type of health care field, or postgraduates practicing in their field. Excluded librarians | Undergraduate and postgraduate (not clearly described) |
| Interventions | Journal clubs. Excluded studies with video/internet and one-off clubs | Journal clubs in different formats |
| Controls | Not described | Not clearly described |
| Outcomes | Learner reaction, attitude, knowledge, skills, behaviour, patient outcomes | Change in reading behaviour; Confidence in ability to critically appraise research; Demonstrated knowledge and critical appraisal skills; Ability to apply findings to clinical practice |
| Date of the most recent search: Not reported | | |
| Limitations: Date of last search not reported; Unclear which databases were searched; Risk of bias assessment of included studies not adequately reported; Characteristics of included studies not clearly described | | |
| Citation: Harris J, Kearley K, Henegan C, Meats E, Roberts N, Perera R, Kearley-Shiers K. Are journal clubs effective in supporting evidence-based decision making? A systematic review. BEME Guide No.16. Medical Teacher 2011; 33:9-23 | | |
