# Supplementary material for: What Are the Effects of Teaching Evidence-Based Health Care (EBHC)? Overview of Systematic Reviews
Source: PLoS One. 2014 Jan 28;9(1):e86706. doi: 10.1371/journal.pone.0086706 (PMC3904944; doi:10.1371/journal.pone.0086706)
Supplement: Table S10 — Characteristics of included systematic review Horsley 2010. (DOCX) [file pone.0086706.s010.docx]

## Table S10. CHARACTERISTICS OF INCLUDED SYSTEMATIC REVIEW HORSLEY 2010

|  | What the review authors searched for | What the review authors found |
| --- | --- | --- |
| Studies | Systematic review of RCTs, CCT, CBA and ITS where there was a clearly defined point in time when the interventions occurred and at least three data points before and after the intervention. | 3 RCT’s; 1 CCT |
| Participants | All health care providers involved in direct patient care. No undergraduates and no students. | Residents; Doctors, nurses, allied health professionals, Occupational health physicians |
| Interventions | Considered interventions designed to increase the frequency and/or quality of healthcare professionals question formulation of any duration and follow-up | Lecture and input from librarian; Live demonstrations, hands on practice sessions; Didactic input, hands-on practice; Questionnaire with written instructions and examples |
| Comparisons | Comparison group could receive no intervention, continued current usual practices or a less intensive intervention. | |
| Outcomes | Primary: Frequency of questions generated; Quality of questions generated; Practitioner competency; Patient delivery of care; Patient-related outcomes; Knowledge-seeking practices; Evidence-based practice(s). Secondary outcomes: Objective measures of self-efﬁcacy; Increased success of answering questions generated; Summary data pertaining to the types of questions generated by healthcare professionals. | Quality of questions; Increased success of answering questions; Knowledge-seeking practices; Self-efficacy; Types of questions generated |
| Date of the most recent search: August 2008 | | |
| Limitations: None | | |
| Citation: Horsley T, O’Neill J, McGowan J, Perrier L, Kane G, Campbell C. Interventions to improve question formulation in professional practice and self-directed learning. *Cochrane Database of Systematic Reviews* 2010, Issue 5. Art. No.: CD007335. DOI:10.1002/14651858.CD007335.pub2. | | |
