# Supplementary material for: What Are the Effects of Teaching Evidence-Based Health Care (EBHC)? Overview of Systematic Reviews
Source: PLoS One. 2014 Jan 28;9(1):e86706. doi: 10.1371/journal.pone.0086706 (PMC3904944; doi:10.1371/journal.pone.0086706)
Supplement: Table S11 — Characteristics of included systematic review Horsley 2011. (DOCX) [file pone.0086706.s011.docx]

## Table S11. CHARACTERISTICS OF INCLUDED SYSTEMATIC REVIEW HORSLEY 2011

|  | What the review authors searched for | What the review authors found |
| --- | --- | --- |
| Studies | RCT’s, controlled clinical trials, controlled before and after studies, interrupted time series (minimum requirement that there has to be a comparison with no teaching in critical appraisal, either in a separate group or in the same group – before teaching. | 3 RCT’s (n=272) |
| Participants | Any qualified healthcare professional (including managers and purchasers) with direct patient care in any given clinical setting. No students | Interns in Internal Medicine, Health care professionals (general practitioners, hospital physicians, professions allied to medicine, and healthcare managers and administrators),Surgeons |
| Interventions | Educational interventions (def: co-ordinated educational activity, of any medium, duration or format) teaching critical appraisal (def: the process of assessing and interpreting evidence by systematically considering its validity, results and relevance to ones’ own work). Single or package of interventions. Teaching of biostatistics and epidemiology excluded | Journal club supported by a half-day workshop (Linzer 1988), critical appraisal materials (package) including papers with methodological reviews, list serve discussions and articles (MacRae 2004) and a half-day workshop based on a Critical Appraisal Skills Programme (CASP) (Taylor 2004). |
| Comparisons | No teaching in critical appraisal, either in a separate group of before intervention | Standard conference series on ambulatory medicine; Access to journals and articles only; waiting list for workshop |
| Outcomes | Objectively measured process of care variables; Objectively measured patient outcomes; Objectively measured assessments of the impact of teaching critical appraisal on health professional’s knowledge/awareness were considered if assessment of outcome measure was based upon standardised and reliable instruments | Knowledge scores; Critical appraisal skills |
| **Date of the most recent search:** January 2010: EMBASE, LISA, ERIC, CDSR, DARE, EPOC specialised register, ISI web of knowledge; June 2011: CENTRAL, MEDLINE | | |
| **Limitations:** No mention of minimising language bias | | |
| **Citation:** Horsley T, Hyde C, SantessoN, Parkes J, Milne R, Stewart R. Teaching critical appraisal skills in healthcare settings. *Cochrane Database of Systematic Reviews* 2011, Issue 11. Art. No.: CD001270. DOI: 10.1002/14651858.CD001270.pub2 | | |
