# Supplementary material for: What Are the Effects of Teaching Evidence-Based Health Care (EBHC)? Overview of Systematic Reviews
Source: PLoS One. 2014 Jan 28;9(1):e86706. doi: 10.1371/journal.pone.0086706 (PMC3904944; doi:10.1371/journal.pone.0086706)
Supplement: Table S12 — Characteristics of included systematic review Hyde 2000. (DOCX) [file pone.0086706.s012.docx]

## Table S12. CHARACTERISTICS OF INCLUDED SYSTEMATIC REVIEW HYDE 2000

|  | What the review authors searched for | What the review authors found |
| --- | --- | --- |
| Studies | Any comparative study design including RCT’s, non RCT’s, CBA’s, interrupted time series, simple before-after designs | 1 RCT; 8 Controlled trials; 7 Before-after studies |
| Participants | Participants in any clinical setting, including health care students, professionals, managers, purchasers, and health care users. | Medical students; Residents; Midwives; Intern doctors; Multidisciplinary (qualified doctors, managers and researchers) |
| Interventions | Educational interventions teaching critical appraisal (single intervention or package). Excluding studies where biostatistics and/or epidemiology were taught | Tutorial; Workshop; Lecture; Seminar; Study day; Journal club |
| Comparisons | Not specified | Not specified |
| Outcomes | **Patient outcomes:** Health outcomes (mortality and morbidity); Quality of life; Satisfaction. **Learner outcomes:**  Behaviour, including process of care; Critical appraisal skills; Knowledge; Attitudes; Satisfaction. **Teacher outcomes:** Satisfaction | Skills; Knowledge; Behaviour; Attitude |
| Date of the most recent search: December 1997 | | |
| **Limitations:** Unclear whether language restrictions were used when searching for studies | | |
| **Citation:** Hyde C, Parkes J, Deeks J, Milne R. Systematic review of effectiveness of teaching critical appraisal. ICRF/NHS Centre for Statistics in Medicine. 2000 | | |
