# Supplementary material for: What Are the Effects of Teaching Evidence-Based Health Care (EBHC)? Overview of Systematic Reviews
Source: PLoS One. 2014 Jan 28;9(1):e86706. doi: 10.1371/journal.pone.0086706 (PMC3904944; doi:10.1371/journal.pone.0086706)
Supplement: Table S13 — Characteristics of included systematic review Ilic 2009. (DOCX) [file pone.0086706.s013.docx]

## Table S13. CHARACTERISTICS OF INCLUDED SYSTEMATIC REVIEW ILIC 2009

|  | What the review authors searched for | What the review authors found |
| --- | --- | --- |
| Studies | Randomized controlled trials and non-randomized trials | 3 RCT’s; 1 CT; 1 (non-randomised) trial; 1 cross-over trial; 1 before after study |
| Participants | Under/postgraduate medical students or under/postgraduate allied health professionals | General practitioners (1 study); Medical residents (1 study); General surgeons (1 study); Undergraduate medical students (2 studies); Undergraduate nursing students (1 study); Naturopathic undergraduate students (1 study) |
| Interventions | EBP teaching: formulating an answerable question, searching medical databases, critical appraisal | Half day EBP workshop (2 studies); 7 week-2hour EBP workshop; EBP multimedia package; Supplemented EBP teaching (directed vs self-directed); 4 EBP tutorials; 2 four-hour EBP workshops |
| Comparisons | Not described | Not described for all studies; Alternative clinical topics; Directed vs. self-directed learning |
| Outcomes | EBP knowledge, skills or behaviour | EBP competency; EBP knowledge, skills and behaviour; Critical appraisal skills; Formulating questions; Searching skills |
| Date of the most recent search: September 2008 | | |
| **Limitations:** Search not comprehensive – did not address language and publication bias; Screening of full texts for inclusion as well as data extraction only done by one author; Results of risk of bias assessment not reported at all; Results of included studies not adequately reported – no measures of effect and CIs reported | | |
| **Citation:** Ilic D. Teaching Evidence-based Practice: Perspectives from the Undergraduate and Post-graduate Viewpoint. Ann Acad Med Singapore 2009;38:559-63 | | |
