# Supplementary material for: What Are the Effects of Teaching Evidence-Based Health Care (EBHC)? Overview of Systematic Reviews
Source: PLoS One. 2014 Jan 28;9(1):e86706. doi: 10.1371/journal.pone.0086706 (PMC3904944; doi:10.1371/journal.pone.0086706)
Supplement: Table S14 — Characteristics of included systematic review Norman 1998. (DOCX) [file pone.0086706.s014.docx]

## Table S14. CHARACTERISTICS OF INCLUDED SYSTEMATIC REVIEW NORMAN 1998

|  | What the review authors searched for | What the review authors found |
| --- | --- | --- |
| Studies | Studies with a control group (excluding before-after designs) | 10 studies: 3 RCTs; 6 CT (1 with cross-over); 1 cohort with historical controls |
| Participants | Undergraduate medical residents or residents | 6 studies involved medical undergraduate students and 4 studies involved residents |
| Interventions | Teaching EBM or critical appraisal | Undergraduate: EBM teaching in internal medicine clerkship (part of course credit) and Residents: Variation of journal club format |
| Comparisons | Not specified | Not specified |
| Outcomes | Measure of performance: knowledge, skill or self-reported use of the literature | Knowledge and skills; Self-reported use of the literature |
| Date of the most recent search: Not clearly stated (searched for studies between 1966 and 1995) | | |
| **Limitations:** Selection criteria did not specify participants and interventions; Authors only searched MEDLINE database. Do not mention whether search was restricted for language and publication status; Authors do not clearly describe the process of selection of studies; Do not provide a list of excluded studies; Unclear how data extraction and risk of bias assessment were conducted; Did not mention heterogeneity and did not address the variability between studies regarding the intervention and the outcomes. | | |
| **Citation:** Norman GR, Shannon SI. Effectiveness of instruction in critical appraisal (evidence-based medicine) skills: a critical appraisal. *Can Med Assoc J* 1998;158:177-81 | | |
