# Supplementary material for: What Are the Effects of Teaching Evidence-Based Health Care (EBHC)? Overview of Systematic Reviews
Source: PLoS One. 2014 Jan 28;9(1):e86706. doi: 10.1371/journal.pone.0086706 (PMC3904944; doi:10.1371/journal.pone.0086706)
Supplement: Table S15 — Characteristics of included systematic review Taylor 2000. (DOCX) [file pone.0086706.s015.docx]

## Table S15. CHARACTERISTICS OF INCLUDED SYSTEMATIC REVIEW TAYLOR 2000

|  | What the review authors searched for | What the review authors found |
| --- | --- | --- |
| Studies | Systematic reviews including studies with a control group | 10 Studies: 1 RCT, 4 non-randomized trials; 3 prospective cohort studies; 1 retrospective cohort study; 1 Cross-sectional study |
| Participants | Health care professionals | Medical students (6 studies) and newly qualified physicians (4 studies) |
| Interventions | Educational intervention of critical appraisal | Educational interventions ranging from a total of 180 min over a 1-week period to 16h over the period of a year |
| Comparisons | No educational intervention or "placebo" educational intervention | No educational input (6 studies); general medical input (2 studies); traditional epidemiological education (2 studies) |
| Outcomes | Educational outcomes and health care outcomes | Knowledge of epidemiology/statistics; Attitudes towards medical literature; Ability to critically appraise an article; Medical literature reading behaviour |
| Date of the most recent search: December 1997 | | |
| **Limitations:** Study selection process unclear; Risk of bias assessment only reported as a score out of 12 – no details of risk of bias for specific domains reported; Vote counting done (according to statistical significance) – no individual results reported | | |
| Citation: Taylor R, Reeves B, Ewings P, Binns S, Keast J, Mears R. A systematic review of the effectiveness of critical appraisal skills training for clinicians. Medical Education 2000; 34:120-5 | | |
