# Supplementary material for: What Are the Effects of Teaching Evidence-Based Health Care (EBHC)? Overview of Systematic Reviews
Source: PLoS One. 2014 Jan 28;9(1):e86706. doi: 10.1371/journal.pone.0086706 (PMC3904944; doi:10.1371/journal.pone.0086706)
Supplement: Table S16 — Characteristics of included systematic review Wong 2013. (DOCX) [file pone.0086706.s016.docx]

## Table S16. CHARACTERISTICS OF INCLUDED SYSTEMATIC REVIEW WONG 2013

|  | What the review authors searched for | | What the review authors found |
| --- | --- | --- | --- |
| Studies | RCTs, CTs or cohort studies (pre-post and longitudinal studies) reporting original data on outcomes evaluating an EBP educational intervention. **Excluded:** Case studies, cross-sectional studies, editorials, narrative and systematic reviews | | 8 included studies - 2 CT; 5 BA; 1 longitudinal study with four test occasions. |
| Participants | Entry-level health professional students. ‘Entry-level’ was defined as undergraduate and graduate entry programs that prepare students to enter their professions as beginning practitioners. | | Entry level: Medical students (n=5); Nursing (n=1); Physiotherapy (n=1); Postgraduate physiotherapy and undergraduate occupational therapy students (n=1); Sample size ranged from 17 to 293 |
| Interventions | At least one EBP educational  intervention which include one or more of the five steps of EBP of any mode of delivery (e.g. lectures, tutorials, online or workshops) or the type of EBP educational interventions (e.g. formal or informal, stand-alone or integrated training). | | Mix of lecture-based and clinically-integrated EBP training covering different steps of EBP. Duration varied from 4 days to 1,5 years. |
| Comparisons | Irrespective of the presence or absence of control groups. | | |
| Outcomes | Self-reported EBP attitudes (value and importance placed on EBP), Behaviours (actual performance and use of EBP in practice), Knowledge (understanding of EBP), Skills (application of EBP knowledge by performing the EBP steps) and Confidence (perception of one’s own ability with EBP skills). | Knowledge, attitudes and skills; All but two studies reported using valid and reliable instruments | |
| Date of the most recent search: December 2011 | | | |
| **Limitations:** Limited to English language articles; Only one reviewer selected studies; Independent data extraction done for a sample of included studies; Risk of bias approach not detailed. All study types assessed in same manner and no specific study related criteria used | | | |
| **Citation:** Wong SC, McEvoy MP, Wiles LK, Lewis LK. Magnitude of change in outcomes following entry-level evidence-based practice training: a systematic review. International Journal of Medical Education. 2013;4:107-14 | | | |
