# Supplementary material for: What Are the Effects of Teaching Evidence-Based Health Care (EBHC)? Overview of Systematic Reviews
Source: PLoS One. 2014 Jan 28;9(1):e86706. doi: 10.1371/journal.pone.0086706 (PMC3904944; doi:10.1371/journal.pone.0086706)
Supplement: Table S17 — Characteristics of ongoing systematic reviews. (DOCX) [file pone.0086706.s017.docx]

## Table S17. CHARACTERISTICS OF ONGOING SYSTEMATIC REVIEWS

|  | RAY 2013 | ROHWER 2013 |
| --- | --- | --- |
| *Studies* | No details provided | Randomized controlled trials and cluster randomized controlled trials will be included. Non-randomized study designs will be excluded. |
| *Participants* | Undergraduate medical students | Health care professionals, including doctors, dentists, nurses, occupational  therapists, physiotherapists, dieticians, audiologists, mental health professionals,  psychologists, counsellors, social workers in any year of postgraduate study with an academic institution. We will not be including continuing professional development activities. |
| *Interventions* | Teaching literature searching skills | A completely web-based (e-learning) module on EBHC, including any or all of the 5 steps of EBHC (Asking questions, searching the literature, critically appraising the literature, applying the results, evaluating the process); integrated into clinical learning or as a stand-alone module; as part of the postgraduate curriculum in the specific field of study |
| *Comparisons* | No details provided | A face-to-face module on EBHC including any or all of the 5 steps of EBHC;  integrated into clinical learning or as a stand-alone module; or a blended module on EBHC consisting of face-to-face and e-learning components; as part of the  postgraduate curriculum in the specific field of study |
| *Outcomes* | Knowledge and skills | *Primary outcomes*: Increased knowledge of EBHC including all or any one of the steps of EBHC (phrasing questions, searching the literature, critically appraising the literature, applying the results, evaluating the process); Improved skills in practicing the steps of EBHC. *Secondary outcomes*: Attitude towards EBHC, measured with a qualitative instrument; Practicing of evidence-based health care in the clinical setting (behaviour); Satisfaction of students with the method of learning; Self-perceived competency in EBHC; Satisfaction of educators with method of teaching |
| *Citation* | Ray A. Is the teaching of literature searching skills for medical students (undergraduates) an effective educational intervention to change their knowledge and skills (title registered with Best Evidence Medical Education (BEME) Collaboration) <http://www.bemecollaboration.org/NewTopics/> | Rohwer A, Young T. E-learning versus face-to-face learning on evidence-based health care (EBHC) for increased EBHC knowledge and skills in postgraduate health care professionals (title registered with Campbell Collaboration) |
