# Supplementary material for: What Are the Effects of Teaching Evidence-Based Health Care (EBHC)? Overview of Systematic Reviews
Source: PLoS One. 2014 Jan 28;9(1):e86706. doi: 10.1371/journal.pone.0086706 (PMC3904944; doi:10.1371/journal.pone.0086706)
Supplement: Table S18 — Matrix of included systematic reviews and the studies included in each. (DOCX) [file pone.0086706.s018.docx]

## Table S18. Matrix of included systematic reviews and the studies included in each

|  | **Primary studies** | | | | | | |  | | **Systematic reviews** | | | | | | | | | | | | | | |
| --- | --- | --- | --- | --- | --- | --- | --- | --- | --- | --- | --- | --- | --- | --- | --- | --- | --- | --- | --- | --- | --- | --- | --- | --- |
|  | *Study ID* | *Type of study* | *Intervention* | *Participants* | *Sample size* | *Outcomes assessed* | *Location* | Ahmandi 2012 [[26](#_ENREF_26)] | Audet 1993 [[27](#_ENREF_27)] | | Baradaran 2013 [[41](#_ENREF_41)] | Coomarasamy 2004 [[28](#_ENREF_28)] | Deenadayalan 2008 [[29](#_ENREF_29)] | Ebbert 2001 [[30](#_ENREF_30)] | Flores Mateo 2007 [[31](#_ENREF_31)] | Green 1999 [[32](#_ENREF_32)] | Harris 2011 [[33](#_ENREF_33)] | Horsley 2010 [[34](#_ENREF_34)] | Horsley 2011 [[35](#_ENREF_35)] | Hyde 2000 [[36](#_ENREF_36)] | Ilic 2009 [[37](#_ENREF_37)] | Norman 1998 [[38](#_ENREF_38)] | Taylor 2000 [[39](#_ENREF_39)] | Wong 2013 [[40](#_ENREF_40)] |
| 1 | Akl 2004 | CT | MI | R,UG | 40 | K, S | USA |  |  | |  |  |  |  | x |  |  |  |  |  |  |  |  | x |
| 2 | Alper 2005 | CBA | MI | UG | 90 | S | USA |  |  | | x |  |  |  |  |  |  |  |  |  |  |  |  |  |
| 3 | Aronoff 2010 | BA | MI | UG | 153 | S | USA |  |  | | x |  |  |  |  |  |  |  |  |  |  |  |  | x |
| 4 | Baum 2003 | BA | SI | R | 73 | A | USA |  |  | |  |  |  |  | x |  |  |  |  |  |  |  |  |  |
| 5 | Bazarian 1999 | CT | SI | R | 32 | K, S, B | USA |  |  | |  | x | X | x | x |  | x |  |  |  |  |  |  |  |
| 6 | Bennett 1987 | CT | SI | UG | 92 | S | Canada |  | x | | x |  |  |  |  |  |  |  |  | x |  | x | x |  |
| 7 | Bennet 2011 | BA | MI | UG | 59 | K, A | Australia |  |  | |  |  |  |  |  |  |  |  |  |  |  |  |  | x |
| 8 | Bolboaca 2006 | CBA | MI | UG | 40 | K | Romania |  |  | | x |  |  |  |  |  |  |  |  |  |  |  |  |  |
| 9 | Bradley 2002 | RCT | SI | R | 10 | S, A, B | USA |  |  | |  | x |  |  |  |  |  | x |  |  |  |  |  |  |
| 10 | Bradley 2005 | RCT | MI | UG | 175 | K, S, A | Norway |  |  | | x |  |  |  |  |  |  |  |  |  | x |  |  |  |
| 11 | Burls 1997 | BA | SI | HP | 1880 | K; A | UK |  |  | |  |  |  |  |  |  |  |  |  | x |  |  |  |  |
| 12 | Cabell 2001 | RCT | MI | R | 48 | S | UK |  |  | |  |  |  |  | x |  |  |  |  |  |  |  |  |  |
| 13 | Caudill 1993 | BA | MI | HP, R | 70 | K, S, A, B | USA |  |  | |  | x |  |  |  |  |  |  |  | x |  |  |  |  |
| 14 | Cheatham 2000 | BA | SI | R | 9 | K | USA | x |  | |  |  |  |  |  |  |  |  |  |  |  |  |  |  |
| 15 | Cheng 2003 | RCT | SI | HP | 800 | K, S, A, B | Hong Kong |  |  | |  |  |  |  | x |  |  | x |  |  |  |  |  |  |
| 16 | Cramer 2001 | BA | SI | R | 35 | K | USA |  |  | |  |  |  |  |  |  | x |  |  |  |  |  |  |  |
| 17 | Cuddy 1984 | BA | SI | UG | 18 | K | USA |  | x | |  |  |  |  |  |  |  |  |  | x |  |  |  |  |
| 18 | Davis 2007 | RCT | SI | I | 55 | K, A | UK |  |  | | x |  |  |  |  |  |  |  |  |  |  |  |  |  |
| 19 | Davis 2008 | RCT | SI | UG | 229 | K, A | UK |  |  | | x |  |  |  |  |  |  |  |  |  |  |  |  |  |
| 20 | Dinkevich 2006 | BA | SI | R | 69 | K, S | USA |  |  | |  |  |  |  | x |  |  |  |  |  |  |  |  |  |
| 21 | Dorsch 2004 | CBA | MI | UG | 36 | A, S | USA |  |  | | x |  |  |  |  |  |  |  |  |  |  |  |  |  |
| 22 | Forsetlund 2003 | RCT | MI | HP | 148 | K, A | Norway |  |  | |  |  |  |  | x |  |  |  |  |  |  |  |  |  |
| 23 | Frasca 1992 | CT | SI | UG | 92 | S | USA |  |  | | x |  |  |  |  |  |  |  |  | x |  | x | x |  |
| 24 | Fritsche 2002 | BA | SI | R,UG | 266 | K, S | Germany | x |  | | x |  |  |  | x |  |  |  |  |  |  |  |  |  |
| 25 | Fu 1999 | CT | SI | R | 24 | K, S, B | UK |  |  | |  | x |  |  | x |  | x |  |  |  |  |  |  |  |
| 26 | Gehlbach 1980 | CT | SI | R | 35 | K | USA |  | x | |  | x |  |  |  | x |  |  |  | x |  | x | x |  |
| 27 | Ghali 2000 | CT | SI | UG | 60 | K, S, B | USA |  |  | |  |  |  |  |  |  |  |  |  |  | x |  |  |  |
| 28 | Grad 2001 | BA | MI | PG | 75 | S, A, B | Canada |  |  | |  | x |  |  |  |  |  |  |  |  |  |  |  |  |
| 29 | Green 1997 | CT | MI | R | 34 | B, K | USA |  |  | |  | x |  |  | x | x |  |  |  |  |  |  |  |  |
| 30 | Gruppen 2005 | CT | SI | UG | 92 | S | USA |  |  | | x |  |  |  |  |  |  |  |  |  |  |  |  |  |
| 31 | Hadley 2010 | RCT | SI | I | 237 | K | UK |  |  | | x |  |  |  |  |  |  |  |  |  |  |  |  |  |
| 32 | Haines 2003 | BA | MI | PG | ? | B | USA |  |  | |  | x |  |  |  |  |  |  |  |  |  |  |  |  |
| 33 | Haynes 1993 | RCT | MI | HP | 392 | S | Canada |  |  | |  |  |  |  | x |  |  |  |  |  |  |  |  |  |
| 34 | Heller 1984 | CT | SI | UG | ? | S | Not stated |  |  | |  |  |  |  |  |  |  |  |  |  |  | x |  |  |
| 35 | Hicks 1994 | BA | SI | HP | 19 | B, S | UK |  |  | |  |  |  |  |  |  |  |  |  | x |  |  |  |  |
| 36 | Hillson 1993 | BA | MI | HP, R | 29 | S | USA |  |  | |  | x |  |  |  |  |  |  |  | x |  |  |  |  |
| 37 | Ibbotson 1998 | BA | SI | HP | 164 | K, S | UK |  |  | |  | x |  |  | x |  |  |  |  | x |  |  |  |  |
| 38 | Johnson 2009 | RCT | SI | UG | 129 | K, A | Hong Kong |  |  | | x |  |  |  |  |  |  |  |  |  |  |  |  |  |
| 39 | Kellum 2000 | BA | MI | HP | 12 | K, S | USA |  |  | |  | x |  |  | x |  | x |  |  |  |  |  |  |  |
| 40 | Khan 1999 | BA | MI | R | 8 | K, A, B | UK | x |  | |  | x | X | x |  |  | x |  |  |  |  |  |  |  |
| 41 | Kim 2009 | CT | MI | UG | 150 | K, A, B | USA |  |  | |  |  |  |  |  |  |  |  |  |  |  |  |  | x |
| 42 | Kitchens 1989 | CT | MI | R | 83 | K | Canada |  | x | |  | x |  |  |  | x |  |  |  | x |  | x | x |  |
| 43 | Krueger 2006 | RCT | MI | UG | 77 | K, S | USA |  |  | | x |  |  |  |  |  |  |  |  |  |  |  |  |  |
| 44 | Kulier 2009 | RCT | SI | R | 61 | K, A | UK and Netherlands | x |  | |  |  |  |  |  |  |  |  |  |  |  |  |  |  |
| 45 | Landry 1994 | CT | SI | UG | 146 | B, K, A | USA |  |  | | x |  |  |  |  |  |  |  |  | x |  | x | x |  |
| 46 | Langkamp 1992 | CT | MI | R | 27 | K | USA |  |  | |  | x | X | x |  | x |  |  |  |  |  |  |  |  |
| 47 | Lai 2009 | BA | MI | UG | 72 | S, K | Malaysia |  |  | | x |  |  |  |  |  |  |  |  |  |  |  |  | x |
| 48 | Lai 2010 | CBA | MI | UG | 65 | A | Malaysia |  |  | | x |  |  |  |  |  |  |  |  |  |  |  |  |  |
| 49 | Lee 2006 | BA | SI | R | 29 | K, S | USA | x |  | |  |  |  |  |  |  | x |  |  |  |  |  |  |  |
| 50 | Lee 2007 | RCT | MI | UG | 155 | K | Hong Kong |  |  | | x |  |  |  |  |  |  |  |  |  |  |  |  |  |
| 51 | Leung 2003 | RCT | MI | UG | 169 | A | Hong Kong |  |  | | x |  |  |  |  |  |  |  |  |  |  |  |  |  |
| 52 | Linzer 1987 | RCT | SI | R | 85 | S | USA |  | x | |  |  | X | x |  |  | x |  |  |  |  | x |  |  |
| 53 | Linzer 1988 | RCT | SI | R | 44 | K, S, B | USA |  | x | |  | x | X | x | x | x | x |  | x | x |  | x | x |  |
| 54 | Lucas 2004 | BA | SI | HP | 33 | B | USA |  |  | |  |  |  |  | x |  |  |  |  |  |  |  |  |  |
| 55 | MacRae 2004 | RCT | MI | HP | 81 | S | Canada |  |  | |  |  | X |  | x |  |  |  | x |  | x |  |  |  |
| 56 | McCluskey 2005 | BA | MI | HP | 114 | K, S, A, B | Australia |  |  | |  |  |  |  | x |  |  |  |  |  |  |  |  |  |
| 57 | McGinn 2002 | RCT | SI | PG | 10 | K, B | USA |  |  | |  | x |  |  |  |  |  |  |  |  |  |  |  |  |
| 58 | McLeod 2010 | RCT | SI | R | 441 | S | USA, Canada | x |  | |  |  |  |  |  |  |  |  |  |  |  |  |  |  |
| 59 | Mills 2002 | CT | SI | UG | 83 | S | Canada |  |  | |  |  |  |  |  |  |  |  |  |  | x |  |  |  |
| 60 | Mulvihill 1981 | BA | SI | PG | ? | K | Not stated |  |  | |  | x |  |  |  | x |  |  |  |  |  |  |  |  |
| 61 | Radack 1986 | CT | SI | UG | 34 | S | USA |  | x | | x |  |  |  |  |  |  |  |  | x |  | x | x |  |
| 62 | Riegelman 1986 | CT | MI | UG | 296 | K, S, B | USA |  | x | |  |  |  |  |  |  |  |  |  | x |  | x | x |  |
| 63 | Romm 1989 | RCT | SI | UG | 108 | K,S | USA |  | x | |  |  |  |  |  |  |  |  |  |  |  |  |  |  |
| 64 | Rosenberg 1998 | RCT | SI | UG | 108 | S | UK |  |  | | x |  |  |  |  |  |  |  |  |  |  |  |  |  |
| 65 | Ross 2003 | CT | SI | R | 48 | K, B | USA |  |  | |  | x |  |  | x |  |  |  |  |  |  |  |  |  |
| 66 | Sastre 2011 | CBA | SI | UG | 100 | B, A | USA |  |  | | x |  |  |  |  |  |  |  |  |  |  |  |  |  |
| 67 | Schaafsma 2007 | CT | MI | HP | 125 | S | Netherlands |  |  | |  |  |  |  |  |  |  | x |  |  |  |  |  |  |
| 68 | Schilling 2006 | RCT | SI | UG | 238 | K, S | USA |  |  | | x |  |  |  | x |  |  |  |  |  |  |  |  |  |
| 69 | Schoenfeld 2000 | BA | SI | PG | 24 | K | USA |  |  | |  | x |  |  |  |  |  |  |  |  |  |  |  |  |
| 70 | Seelig 1991 | BA | SI | R | 14 | K, S, A, B | USA |  |  | |  | x | X |  |  | x | x |  |  | x |  |  |  |  |
| 71 | Seelig 1993 | CT | SI | I | 30 | K, S, A, B | USA |  |  | |  | x |  |  |  |  |  |  |  | x |  |  |  |  |
| 72 | Smith 2000 | CT | SI | R | 55 | K, S, B | USA |  |  | |  | x |  |  | x |  |  |  |  |  | x |  |  |  |
| 73 | Stevermer 1999 | RCT | SI | R | 59 | K, S | USA |  |  | |  |  |  |  | x |  |  |  |  |  |  |  |  |  |
| 74 | Straus 2005 | BA | MI | R | 47 | P | UK |  |  | |  |  |  |  | x |  |  |  |  |  |  |  |  |  |
| 75 | Taheri 2008 | BA | SI | UG | 24 | K, S | Iran |  |  | | x |  |  |  |  |  |  |  |  |  |  |  |  | x |
| 76 | Taylor 2004 | RCT | SI | HP | 145 | K, S, A, B | UK |  |  | |  | x |  |  | x |  |  |  | x |  | x |  |  |  |
| 77 | Toedter 2003 | BA | MI | R | 14 | K, S | USA | x |  | |  |  |  |  |  |  |  |  |  |  |  |  |  |  |
| 78 | Villanueva 2001 | RCT | SI | HP | 52 | S, B | Australia |  |  | |  |  |  |  | x |  |  | x |  |  |  |  |  |  |
| 79 | Vinegra 1986 | CT | MI | UG,R | 47 | K | Mexico |  |  | |  |  |  |  |  |  |  |  |  |  |  |  | x |  |
| 80 | Weberschock 2005 | CBA | SI | UG | 132 | K, S | Germany |  |  | | x |  |  |  |  |  |  |  |  |  |  |  |  |  |
| 81 | West 2011 | CBA | MI | UG | 99 | K, S | USA |  |  | | x |  |  |  |  |  |  |  |  |  |  |  |  |  |

SI – Single Intervention UG- Undergraduate K – Knowledge BA – Before After study

MI – Multifaceted intervention R – Residents A – Attitude CBA – Controlled Before

After study

## I – Interns S – Skills CT – Controlled Trial

## HP – Health B – Behaviour RCT – Randomized

## Professionals Controlled Trial

# P – Practice
